# The Effect of Free-Field Presentation and Processing Strategy on a Measure of Spectro-Temporal Processing by Cochlear-Implant Listeners

**DOI:** 10.1177/2331216520964281

**Published:** 2020-12-11

**Authors:** Alan W. Archer-Boyd, Tobias Goehring, Robert P. Carlyon

**Affiliations:** Cambridge Hearing Group, MRC Cognition and Brain Sciences Unit, 2152 University of Cambridge, Cambridge, United Kingdom

**Keywords:** cochlear implant, spectro-temporal, free-field, behavioral test

## Abstract

The STRIPES (Spectro-Temporal Ripple for Investigating Processor EffectivenesS) test is a psychophysical test of spectro-temporal resolution developed for cochlear-implant (CI) listeners. Previously, the test has been strictly controlled to minimize the introduction of extraneous, nonspectro-temporal cues. Here, the effect of relaxing many of those controls was investigated to ascertain the generalizability of the STRIPES test. Preemphasis compensation was removed from the STRIPES stimuli, the test was presented over a loudspeaker at a level similar to conversational speech and above the automatic gain control threshold of the CI processor, and listeners were tested using the everyday setting of their clinical devices. There was no significant difference in STRIPES thresholds measured across conditions for the 10 CI listeners tested. One listener obtained higher (better) thresholds when listening with their clinical processor. An analysis of longitudinal results showed excellent test–retest reliability of STRIPES over multiple listening sessions with similar conditions. Overall, the results show that the STRIPES test is robust to extraneous cues, and that thresholds are reliable over time. It is sufficiently robust for use with different processing strategies, free-field presentation, and in nonresearch settings.

Most cochlear-implant (CI) listeners understand speech in quiet, though performance can vary greatly. In noisy situations, even high-performing listeners will struggle to understand speech. Many new ways of improving CIs have been developed to increase the number of listeners who might benefit, particularly when listening to speech in noise. The methods range from new stimulation modes (Arora et al., 2011; Bierer & Litvak, 2016; Donaldson et al., 2005; Holden et al., 2002; Litvak, Spahr & Emadi, 2007; Litvak, Spahr, Saoji, et al., 2007; van den Honert & Kelsall, 2007), to novel processing strategies (Goehring et al., 2017, Goehring, Archer-Boyd, et al., 2019; Goehring, Keshavarzi, et al., 2019; Loizou et al., 2000; Riss et al., 2008; van Hoesel et al., 2008) and improvements in the way devices are tailored to individual patients (Garadat et al., 2012; Noble et al., 2014). Suitable tests to quickly and accurately assess these methods have also been sought, as speech tests rely heavily on a listener’s acclimatization to their processing strategy over timescales that are much longer than a clinical appointment (Davis & Johnsrude, 2007; Davis et al., 2005). The acclimatization required with speech tests complicates the evaluation of novel strategies when compared with the listener’s clinical strategy, as the latter has a systematic advantage due to long-term usage. The potentially limiting factor of acclimatization has been discussed in several recent studies that used speech to evaluate novel processing strategies (Berg et al., 2019; Croghan et al., 2017; Schvartz-Leyzac et al., 2017). In the worst case, this can lead to false conclusions and the prevention of further investigation into potentially beneficial new methods or strategies. Take-home experience with a new program is a valid solution, but one that, if the program is ultimately unsuccessful, may involve subjecting the listener to long periods of poor hearing.

Many nonspeech tests have been developed in order to circumvent the need for take-home testing, with the aim of providing quick, reliable information on whether a listener’s auditory perception has been improved by a manipulation of a CI program (Aronoff & Landsberger, 2013; Azadpour & McKay, 2012; Drennan et al., 2008; Henry & Turner, 2003; Litvak, Spahr, Saoji, et al., 2007; Saoji et al., 2009; Supin et al., 1994; Won et al., 2007, 2011). We have previously described a number of criteria that such tests should meet (Archer-Boyd et al., 2018). These include the necessity for the listener to perform both spectral *and* temporal processing to obtain good performance, and the related requirement that performance should not be possible based on any local spectro-temporal segment. Instead, the test should require a higher order cue to be extracted from the stimuli such as the direction of frequency sweeps (Archer-Boyd et al., 2018). A nonspeech, truly spectro-temporal test avoids the learning effects and translation requirements inherent in speech tests, while measuring an important factor in speech intelligibility, namely, spectro-temporal processing. Here, spectro-temporal processing is defined as requiring a combination of information across both frequency and time, rather than simply requiring the listener to monitor one frequency channel or to perform an instantaneous across-frequency judgment. One test that fulfills these criteria is the spectro-temporal ripple for investigating processor effectiveness (STRIPES) test (Archer-Boyd et al., 2018). The STRIPES test presents listeners with concurrent sinusoids that either decrease or increase in frequency and requires them to discriminate between upward- and downward-sweeping stimuli (cf. Figure 1

**Figure 1.**
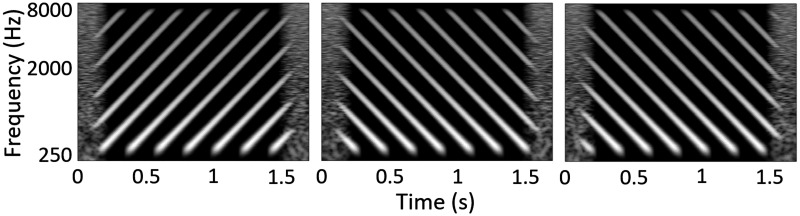
Spectrograms of the STRIPES Stimuli at Density = 5 (1 RPO/5 Hz AM). The left plot shows up STRIPES, and the middle and right panels show two down STRIPES at different starting phases.

). Difficulty is increased by increasing the temporal overlap between each sweep. Although the amplitude modulation (AM) in any one frequency region changes with stripe density, it remains the same for both up and down sweeps. The STRIPES test has been shown to be sensitive to large reductions in spectral resolution at the level of individual listeners, and the test was simple enough to obtain thresholds from newly implanted listeners (Archer-Boyd et al., 2018). The test also successfully predicted which of two experimental fitting algorithms, each of which deactivated different subsets of electrodes, produced the best speech-in-noise performance at the level of individual listeners (Goehring Archer-Boyd, et al., 2019; Goehring, Keshavarzi, et al., 2019) and was correlated with an individual measure of channel interaction based on spectrally blurred speech-in-noise stimuli (Goehring et al., 2020).

STRIPES was initially developed for the HiRes-S strategy implemented in CIs manufactured by Advanced Bionics, and great care was taken to remove any within-channel cues (at least, those visible in the electrodograms) that could be used to perform the task based on cues other than spectro-temporal modulation. Stimuli were presented at a level below the automatic gain control threshold (AGC) via a direct-audio input from a soundcard to the CI processor, removing possible confounding factors such as room reverberation, loudspeaker/microphone frequency response, preemphasis processing, and spectral filtering due to head movement. As much additional processing of the stimuli (e.g., noise reduction) was disabled as possible. In comparison, the majority of research and clinical auditory tests are presented in free-field via a loudspeaker, at a sound level similar to normal conversational speech (60–70 dB SPL, above the threshold of most AGCs), and identical stimuli are used across manufacturers (e.g., Aronoff & Landsberger, 2013; Kollmeier et al., 2015; Spahr et al., 2012). Input is via a microphone on the CI processor, and the listener’s everyday clinical program is often used, meaning that additional processing such as noise-reduction algorithms may remain active and affect the presented stimuli.

All of these factors make testing easier in the clinical setting, but potentially introduce extraneous cues that could be used to perform the task. Indeed, spectral and spectro-temporal ripple tests such as the spectral–temporally modulated ripple test by Aronoff and Landsberger (2013) can contain cues that allow the task to be performed using either purely within-channel temporal processing or by comparing the across-channel profile of excitation in a single short time segment—hence not meeting our proposed requirement for truly spectro-temporal processing. As noted earlier, a spectro-temporal cue is defined as any cue that requires comparison across time *and* frequency. Nonspectro-temporal cues, which require only temporal *or* spectral processing, could include within-channel AM cues at low ripples-per-octave (Anderson et al., 2012; Archer-Boyd et al., 2018), and spectral aliasing due to the number and spacing of the filter bands used in the vocoder or CI (conference abstract, O’Brien & Winn, 2017). These issues are less of a problem for the STRIPES test, for which psychometric functions are monotonic for CI listeners (Archer-Boyd et al., 2018). A study using stimuli and methods that are similar to the STRIPES test has found no evidence of nonspectro-temporal cues and has found that performance can be predicted by the highest performing octave band, not by average broadband performance in normal-hearing and hearing-impaired listeners (Narne et al., 2018).

The work presented here quantifies the effect on listener performance of easing the previously strict experimental conditions and thus potentially making the test more flexible and applicable to a clinical setting. To do so, the effect of presenting the stimuli via loudspeaker instead of the direct auditory input was examined. The use of the patient’s own clinical processing strategy—rather than the HiRes-S strategy used for developing STRIPES—was also examined.

All listeners used the HiRes Optima-S clinical strategy in their everyday settings. In previous STRIPES studies, the experimental maps used the HiRes-S strategy. One major difference between these strategies is that Optima uses current steering between electrodes. Current steering potentially increases the number of channels (or virtual channels) that a listener can discriminate (Firszt et al., 2007; Koch et al., 2007; Landsberger & Srinivasan, 2009). However, speech intelligibility results have been mixed when using current steering across the whole array versus no steering (Firszt et al., 2009; Mens & Berenstein, 2005). The Optima-S strategy limits current steering such that the proportion of current applied to the more apical electrode of each pair ranged between 0.25 and 0.75 (Advanced Bionics, 2013).

This study compares the performance on different implementations of the STRIPES test. One of these is the original method using direct auditory input and the HiRes-S strategy, reported by Goehring, Archer-Boyd, et al. (2019), whose data are reproduced here. The second uses the same HiRes-S strategy but presents the stimuli via loudspeaker. Finally, loudspeaker presentation is used, combined with the listener’s clinical (HiRes Optima-S) strategy. The data from all three conditions were obtained from the same listeners.

## Methods

### Overview of the STRIPES Test

The STRIPES test procedure is identical to the implementation described by Goehring, Archer-Boyd, et al. (2019) and is described briefly here. The test uses an adaptive procedure to measure the spectral-density threshold at which the listener can just distinguish the target stimulus from two reference stimuli in a three-interval, two-alternative forced-choice task. Stimuli are created from 1 s-long, concurrent, and concatenated exponential sine sweeps (with random starting phase) moving up or down in frequency from 250 to 8000 Hz, at a rate of 5 octaves per second. The delay between the start of each sweep is 1/density, such that at density = 5, the delay between the start of each sweep is 200 ms. As the bandwidth of the stimulus is 5 octaves, the number of ripples per octave (RPO) is equal to density/5. As the individual sweeps are 1 s in duration, the AM frequency in Hz is the same as the density. A single cycle of a STRIPES stimulus can be described in the discrete time domain by the following equation, after Berdahl and Smith (2008),

$$\begin{array}{l} s\left(n\right)=\overset{D}{\underset{d\in \mathbb{Z}\cap^{\text{​}}\left[0,D\right]}{\sum }}\sin\left[K\left(e^{\frac{-\left(n+\left(\frac{df_{s}}{D}\right)\right)}{Lf_{s}}}-1\right)\right]\\ \;\text{if}\;n+\left(\frac{df_{s}}{D}\right)\leq Tf_{s},\;\text{for}\;n=1,\ldots ,\frac{f_{s}}{D} \end{array}$$

where *n* = sample, *f_s_* = sample rate (=48kHz), *D* = density, *d* = individual sweep, *T* = sweep duration (=1 s), $K=\frac{\omega_{1}T}{ln\left(\frac{\omega_{2}}{\omega_{1}}\right)}$, and $L=\frac{T}{ln\left(\frac{\omega_{2}}{\omega_{1}}\right)}$.

The number of cycles, and hence the duration of the stimuli, decreases with increasing density. This was in order to ensure that at least two unbroken single sweeps are presented in each stimulus regardless of the starting phase, and that an integer number of cycles at each density is presented. The duration of the STRIPES stimuli varied from 2.07 s (density = 1.1) to approximately 1.3 s at the highest densities presented. Ensuring an integer number of cycles also has the effect of matching the start and end instantaneous frequencies for each stimulus, minimizing this potentially confusing and salient cue. The stimuli used for loudspeaker presentation were identical to those used in the normal hearing (NH) vocoder experiment (Experiment 1) of Archer-Boyd et al. (2018). The changes from the previous CI version of the stimuli were the removal of preemphasis compensation, and the application of equal 50-ms onset/offset ramps to each individual sweep in the stimuli.

The preemphasis compensation was initially added to the stimuli to reduce the effect of the high-pass preemphasis filter used in AB devices and described in Boyle et al. (2009). However, preemphasis does not introduce any additional cues that would allow the listener to perform the STRIPES task and may result in the low-frequency channels being higher in level than in everyday listening. Therefore, in this version, preemphasis compensation was removed. Previously, the duration of the ramps imposed to smooth the onsets and offsets of the glides differed depending on whether each glide ended at a high (8000 Hz) or a low (250 Hz) frequency. This was done in order to make the individual sweep envelopes more symmetric in the lowest and highest channels where the sweeps started and ended. However, this symmetry changed depending on the filters used and would not be practical for testing across strategies, so standard, equal-duration (50 ms) raised cosine ramps were applied to the starts and ends of all sweeps.

The listener selects the target interval, which is either the first or last interval, and which is always an upward sweep; the other two intervals contain downward sweeps. The starting phase of the stimuli is randomized. The number of concurrent frequency sweeps (the density) is varied to titrate difficulty, with the task being easy at a density close to one peak per 5 octave sweep, and progressively harder at higher densities. Note that noninteger density values are possible; for example, a density of 2.5 would mean that 50% of the time two swept sinusoids were present simultaneously (overlapped) and that for the other 50% of the time three swept sinusoids overlapped. Example spectrograms of the stimuli at density 5 (1 RPO and 5 Hz AM) are shown in Figure 1. The starting frequency is roved across trials and the beginning and end of each interval are masked by short noise bursts to reduce the salience of onset and offset cues. An adaptive two-up/one-down procedure starts with a sweep density of 1.1 (number of sweeps concurrently presented during each sweep) and adjusts the density per trial with a density step size of 0.5 (for the first four reversals) and 0.2 (for the last eight reversals). The interstimulus interval was 0.6 s. The test ends after 12 reversals and the final threshold of the run was calculated as the average of the last 4 reversals.

### Apparatus

Listeners took part in the STRIPES test in a double-walled Industrial Acoustics Company (IAC Acoustics, Winchester, UK) sound-proof booth measuring 2 × 2 × 2 m. A Dell Inspiron laptop (Dell Inc., Round Rock, TX) was used to present the stimuli through an RME Fireface UCX (RME, Haimhausen, Germany) external soundcard connected to a Genelec 8030 C loudspeaker (Genelec Oy, Iisalmi, Finland). The loudspeaker had a flat (±2 dB) response from 250 to 8000 Hz (the bandwidth of the stimuli). The loudspeaker was placed 0.75 m from the wall opposite the door, and 0.35 m from the wall adjacent to the door, at a height of 1.2 m to the center of the loudspeaker. The loudspeaker was deliberately placed off-axis in the room, in order to minimize the effect of room modes. An office chair was placed in front of the speaker, such that the listener’s ear was approximately 1 m from the loudspeaker. The stimuli were presented at a level of 65 dB SPL. This level was calibrated using a sound-level meter 1 m from the loudspeaker at a height of 1.2 m, using a noise with the same long-term spectrum and root mean square (RMS) as the STRIPES stimuli. As a final check, the levels of up and down STRIPES at a density of 10 were also measured at the same position.

### Conditions

Table 1

**Table 1.** Differences Between the Experimental Conditions.

Condition	Device/CI program	Presentation	Preemphasis compensation	Sweep onset/offset ramps (ms)	No. participants
Clinical HiRes Aux 1 & 2	Clinical Harmony™/HiRes-S	Auxiliary input	Yes	100/25	8
Research HiRes Aux	Research Harmony^™^/HiRes-S	Auxiliary input	Yes	100/25	10
Research HiRes LS	Research Harmony^™^/HiRes-S	Loudspeaker/omni-mic	No	50/50	10
Clinical Optima LS	Clinical Naida^™^/Optima-S	Loudspeaker/T-mic	No	50/50	10

shows the differences between all experimental conditions. Clin. HiRes Aux 1 & 2 were identical conditions, tested in two different sessions. Clinical (Clin) means the processor is the same as those used in the clinic and programmed using the clinical fitting program Soundwave 3 (Advanced Bionics, 2019). High resolution (HiRes) is AB’s older continuously interleaved sampling (CIS) strategy, without current steering. Auxiliary (Aux) input is the direct audio input on the Harmony™ that bypasses the microphone input and presents audio directly from a soundcard. Rsrch. HiRes Aux was the same condition tested using a research Harmony™ processor instead of a clinical Harmony™ processor (see Table 3 for the differences between the devices). The research processor was programmed using the Bionic Ear Programming System Plus, or Bionic Ear Programming System plus (BEPS+) (Advanced Bionics, 2014). The main difference between the research and clinical Harmony™ is the filters, and these will be described in greater detail in the hardware and processing differences section. The results from these conditions were previously presented in Goehring, Archer-Boyd, et al. (2019). Rsrch. HiRes LS used a research Harmony™ processor and a different version of the STRIPES stimuli (detailed in Overview of the STRIPES test, Methods), presented over a loudspeaker (LS). Clin. Optima LS used the same presentation method and test stimuli as Res. HiRes LS, and the listener used their clinical device. Optima is the current-steering CIS strategy currently used by default in clinical AB devices. The strategy uses pairs of electrodes for each channel, delivering between 25% and 75% of the current to each electrode in a pair.

### Listener Demographics and CI Processor Settings

Ten experienced CI listeners, all of whom were unilateral users of an Advanced Bionics implant, took part. The demographic information of the listeners is given in Table 2

**Table 3.** Comparison of Clinical and Research Harmony™ With HiRes-S and Naida™ With Optima-S.

Hardware/Processing	Clinical Harmony™/HiRes-S	Research Harmony™/HiRes-S	Clinical Naida™/Optima-S
Input/Microphone	Auxiliary input; Omni-dir.; Front behind-the-ear (BTE)-type	Auxiliary input; Omni-dir.; Front behind-the-ear (BTE)-type	Omni-dir.; in-ear T-mic
Channel filters	Time domain	Frequency domain; Fourier transform (FT) based	Frequency domain; Fourier transform (FT) based
Current steering	None	None	Max. 75%/Min. 25%
Max. Voltage (V)	8	8	4

**Table 2.** Listener Demographic Information.

Listener	Sex	Age (years)	Duration implanted	Duration since onset of profound hearing loss	Etiology/acquired pre-/postlingual	CI speech processor	Implant type/CI electrode array	Clinical strategy	Pulse width (µs)	Electrodes deactivated
AB1	M	74	10	41	Unknown/postlingual	Naída CI Q90	HR90K/HiFocus 1 J	HiRes Optima-S/ClearVoice medium	26.0	16
AB2	F	60	11	27	Possible otoxicity/postlingual	Naída CI Q90	HR90K/ HiFocus 1 J	HiRes Optima-S/ClearVoice medium	35.0	16
AB3	M	72	11	36	Otosclerosis/postlingual progression	Naída CI Q90	HR90K/ HiFocus 1 J	HiRes Optima-S/ClearVoice off/CRoS	30.5	12
AB6	F	70	5	65	Unknown/peri-lingual	Naída CI Q90	HR90K/ HiFocus 1 J	HiRes Optima-S/ClearVoice high	35.0	16
AB19	M	75	3	–	Unknown/postlingual progression	Naída CI Q90	HR90k Advantage/ HiFocus MS	HiRes Optima-S/ClearVoice medium/hearing aid removed	34.1	None
AB20	M	74	3	>30	Unknown/postlingual progression	Naída CI Q90	HR90k Advantage/HiFocus MS	HiRes Optima-S/ClearVoice medium	29.6	None
AB23	F	60	3	58	Unknown/peri-lingual	Naída CI Q90	HR90k Advantage/HiFocus MS	HiRes Optima-S/ClearVoice medium	24.2	None
AB24	F	49	3	4	Unknown/postlingual sudden	Naída CI Q90	HR90k Advantage/HiFocus MS	HiRes Optima-S/ClearVoice off	34.1	15, 16
AB25	F	66	3	–	Unknown/postlingual	Naída CI Q90	HR90k Advantage/HiFocus MS	HiRes Optima-S/ClearVoice medium	18	16
AB26	F	58	5	–	Unknown/postlingual	Naída CI Q90	HR90k Advantage/HiFocus MS	HiRes Optima-S	22.4	None

*Note*. CI = cochlear-implant; CROS = contralateral routing of signal.

. Ethical approval was obtained from the National Research Ethics Committee for the East of England. Before commencing the experiments listeners gave their informed consent to participate and were informed that they could withdraw from the study at any point. Listeners were paid for taking part and travel expenses were reimbursed. Listeners’ clinical settings varied, with different levels of ClearVoice™ noise reduction applied. ClearVoice™ aims to identify channels that do not contain speech energy and reduce the gain applied to them in order to enhance the signal-to-noise ratio (Advanced Bionics, 2012). One listener used a linked contralateral routing of signal device. All listeners’ everyday clinical programs were HiRes Optima-S™.

### Test Procedure

Listeners were seated in the booth, and pseudo-randomly assigned to first listen through either their own clinical processor set to everyday program (Clin. Optima LS) or through the Research Harmony™ processor, running a HiRes version of their map with no further signal processing enabled (Rsrch. HiRes LS). Half started with their clinical device. The levels of the STRIPES stimuli were gradually increased to the required 65 dB SPL. The listeners were asked to rate the level of the stimuli on an 11-point loudness scale ranging from 0 (inaudible) to 10 (uncomfortably loud). A rating of 6 (most comfortable) was required. To obtain this rating for listeners AB3 and AB19, the level had to be further increased by adjusting the volume control on the device. The STRIPES test was then explained to the listeners, and the experimenter exited the booth and closed the doors before the test was started by the listener.

The listeners were presented with five pretest forced-choice trials, at density = 1.1 (easiest), in order to familiarize themselves with the STRIPES test. The pretest was repeated until the listener achieved at least 4/5 correct trials; all listeners achieved this on their first attempt. After successful completion of the pretest, the listeners completed one run of the adaptive track and raised their hand to indicate that the test was complete. The experimenter reentered the booth and noted the threshold for the run. After two runs, if the thresholds were different by >1 density, a third run was performed, and the scores from all three were averaged. After completion of two or three runs with one device and program setting, the listener was fitted with the other device and program setting, and the same procedure was carried out. In total, between four and six thresholds were measured for each listener in their first and/or only session. Each run took approximately 8.5 min (*SD* 1.3 min). The duration of each session was 45 min to 1 hr.

### Hardware and Processing Differences

The experimental conditions differed both in the processing strategy used and in the processor hardware, which was either a laboratory-owned clinical or research Harmony™ processer in the HiRes-S conditions, or the listener’s own Naida™ processor in the Optima-S conditions. Key differences and similarities between the three device/processing types are shown in Table 3. One specific difference to note concerns the filters used in the clinical Harmony™ versus the research Harmony™ and clinical Naida™ processors. The frequency domain filters in the research Harmony™ and clinical Naida™ processors use a 128-point Fourier transform, which is separated into bands for envelope extraction. Electrical output of STRIPES when viewed on an oscilloscope was markedly different between the two devices in the low frequencies. The time-domain filters were sharper than the frequency domain filters in the first two to three channels, depending on frequency allocation. This parameter may have affected STRIPES performance as the filter roll-off changed the shape (essentially, the AM) of the envelope in the low-frequency channels. The Naida™ uses a sequential, current-steered CIS strategy (Optima-S™), whereas the Harmony does not use current steering (HiRes-S™). Current steering means that all electrodes stimulate in pairs. In the Optima strategy, it is constrained so that at least 25% of the total charge is presented to one electrode, and no more than 75% of the charge presented to the other.

## Results

**Figure 2.**
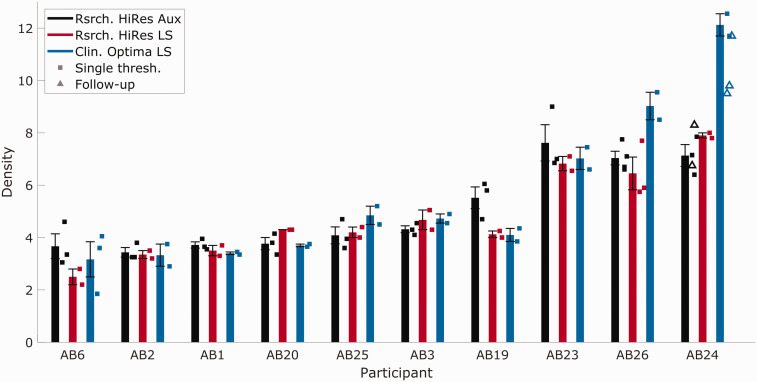
Mean and Single STRIPES Thresholds for Individual Participants. The black bars and squares show data from the original STRIPES test with a HiRes-based program and the same device across participants, reproduced from Goehring, Archer-Boyd, et al. (2019). Red bars/squares show the mean/single thresholds for the same device/program as before, but with STRIPES presented over a loudspeaker. Blue bars/squares show thresholds measured using the same loudspeaker-presented STRIPES test and the participant’s clinical device, set to their everyday program. Black and blue triangles show thresholds collected during additional sessions investigating AB24’s high performance and are not included in the average and standard errors.

Data from the free-field HiRes-S and Optima S conditions are shown by the red and blue bars, respectively, in Figure 2

; data from Goehring, Archer-Boyd, et al. (2019) obtained with the previous version of the STRIPES test (e.g., preemphasis compensation, presentation below AGC threshold, etc.), using the direct (i.e., auxiliary) input and HiRes-S strategy are shown by the black bars for comparison. A one-way repeated-measures analysis of variance showed no effect of condition, *F*(2, 27) = 0.31, *p* = .7343. Hence there is no indication that, overall, changing to loudspeaker presentation or using the patient’s clinical device and strategy either improved or degraded performance. One listener, AB24, performed markedly better with their clinical Naida processor and Optima-S strategy. AB26 showed a smaller possible improvement. Subsequent sessions to find the source of AB24’s high performance were inconclusive (not shown).

## Discussion

### Comparability and Replicability of STRIPES Implementations

**Figure 3.**
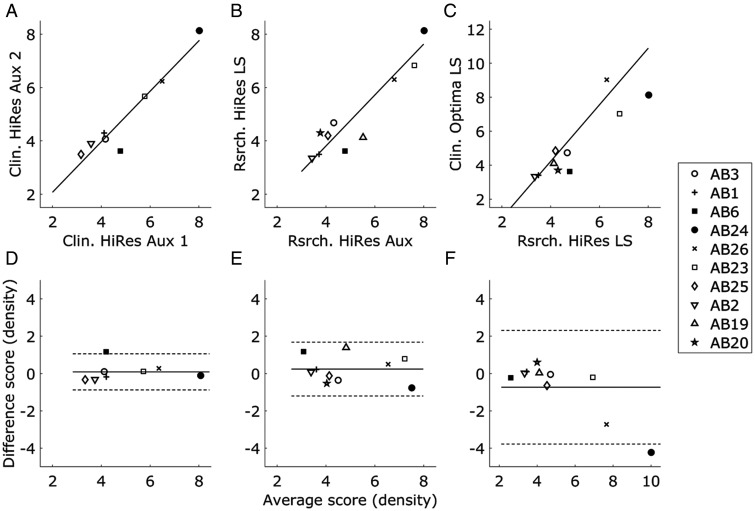
STRIPES Performance for Three Comparisons. The top row shows the correlations between thresholds in each pair of conditions with regression lines (solid lines), and the bottom row shows the differences between thresholds plotted against the average in thresholds with the mean of differences (solid lines). (A and D) Test–retest results based on data from Goehring, Archer-Boyd, et al. (2019) who used the same experimental setup and test conditions and serves as baseline (*N* = 8). (B and E) Comparison of STRIPES thresholds between auxiliary and loudspeaker presentation using a research Harmony™ processor (*N* = 10). (C and F) Comparison of STRIPES thresholds between a research Harmony™ processor with a HiRes program and a clinical Naida™ processor with a Optima program, using loudspeaker presentation. The dotted lines on the bottom row plots indicate the ± two *SDs* from the mean of differences.

As noted in the Results section, there was no significant difference in overall performance between the three different implementations of the STRIPES test. Of greater interest is the extent to which important information about the variation in spectro-temporal sensitivity is preserved when switching to loudspeaker presentation and from the HiRes-S to the clinical strategy. One common method for assessing agreement between two clinical tests is to measure the across-listener correlation between them. A high correlation shows that the two tests capture the same underlying variability across listeners but does not necessarily show that they agree—for example, one test could consistently provide thresholds double that of the other. An alternative method was recommended by Bland and Altman (1986). That method is first illustrated here with reference to the data of Goehring, Archer-Boyd, et al. (2019) who measured STRIPES thresholds for three maps twice on two different days—essentially a test–retest experiment. This provides a “gold standard” against which to test agreement between conditions (*N* = 8). Depicted in Figure 3

A, the across-listener Pearson correlation between the two sessions (Clin. HiRes Aux 1 and 2) was strong (*r* = .96, *p* < .001, *df* = 7). Bland and Altman recommend first plotting the difference between the two sessions as a function of their mean, which is done in Figure 3D, showing that the error is roughly constant across different levels of performance (*r* = .03, *p* = .94, *df* = 7) and that there is no substantial bias (mean difference = 0.09). They note that 95% of points should fall within ±1.96 standard deviations (*SD*), which is indicated by the dotted lines and where the *SD* corresponds to a density of 0.49. It can also be seen that 95% of points show an absolute deviation of less than 1 density unit. This provides some support for the practice of obtaining a third run when the density thresholds from the first two differ by more than density = 1 (0.2 RPO/1 Hz AM).

Performance in the HiRes-S condition with direct input versus loudspeaker presentation is shown in Figure 3B (Rsrch. HiRes Aux and Rsrch. HiRes LS) and correlated strongly for the listeners in this experiment (*r* = .91, *p* < .001, *df* = 9). The Bland–Altman method (Figure3E) shows no evidence that the error covaried with the mean (*r* = −.12, *p* = .74, *df* = 9) nor that there was a bias (mean difference = 0.24), and a *SD* of 0.72 which is only moderately higher than the test–retest reliability (Figure 3D). The correlation between the two loudspeaker conditions (Rsrch. HiRes LS and Clin. Optima LS) was *r* = .91 when listener AB24’s data were excluded and *r* = .94 when they were included (with *p* < .001, *df* = 9, for both). Again, there was no evidence that the error correlated with overall performance (Spearman’s rho = −0.6, *p* = .07, *df* = 9; the error was not normally distributed as determined by the Anderson–Darling test with *p* < .001). There was also no evidence for a substantial bias (mean difference of −0.35 when excluding AB24 and −0.73 for all listeners). The *SD* of errors was 0.95 without listener AB24 and 1.5 for all listeners (Figure 3F).

Overall, the STRIPES test appears robust to manipulations that make it easier to use clinically; a common pattern of variation is observed across listeners, and there is no consistent increase or decrease in performance. Hence, it appears that both the overall level of performance and the variation across listeners are generally impervious to cues that may arise from the AGC, processing strategy, room effects, loudspeaker/microphone frequency response, and spectral filtering due to (small) head movements. However, it is important to understand what cues are available to the listeners in each condition. The next subsection performs such a comparison for the HiRes-S and Optima-S processing strategies. This comparison is of particular interest given that the largest difference observed between any pair of conditions in the experiment was between these two processing strategies, with listener AB24 showing unusually high performance with the Optima S strategy.

### Comparison Between HiRes-S and Optima-S Conditions

**Figure 4.**
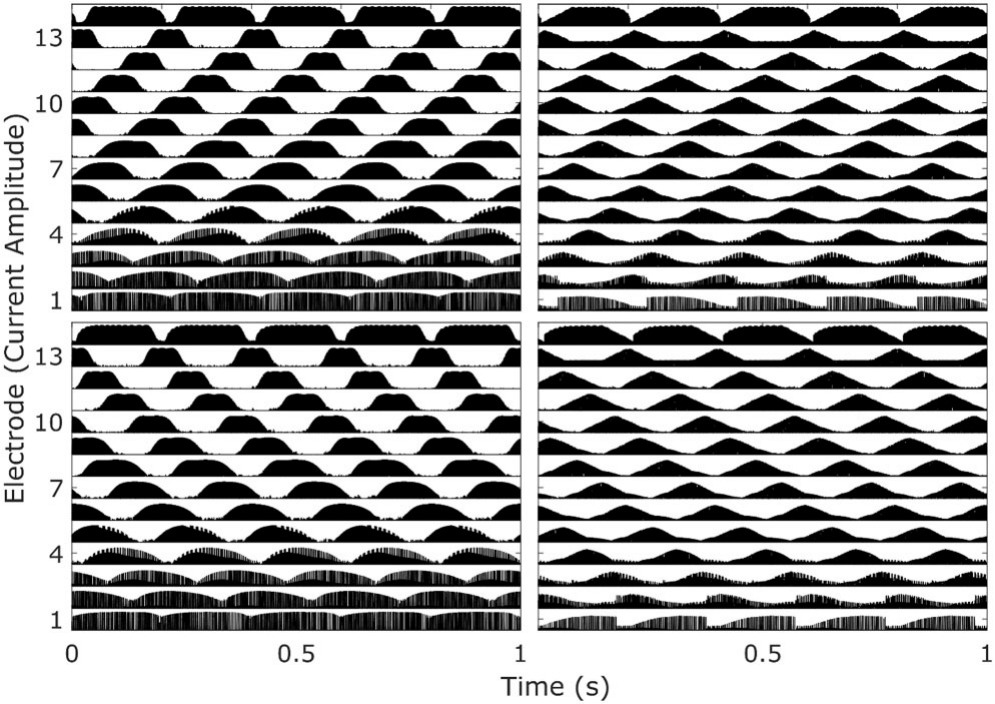
Electrodogram Plots of Density = 5 STRIPES Stimuli When Processed Using AB24’s HiRes (Left Panel) and Optima (Right Panel) Programs. The top row shows UP STRIPES, and the bottom row shows DOWN STRIPES.

The left-hand column of Figure 4

shows electrodograms for the HiRes-S strategy at a density of 5, for up glides (top row) and down glides (bottom row). This was produced using the BEPS+ MATLAB simulator, meaning that it uses the research Harmony™ frequency domain filters. This density is close to the average threshold across all listeners shown in Figure 2 (color online). The directions of the glides are clearly visible and, importantly, there is no clear cue in any one channel that would allow the listener to perform the task. Analogous plots for the Optima-S strategy are shown in the right-hand column. Again, the directions of the glides are visible. A difference between the two strategies is that the envelope peaks are sharper for Optima-S than for HiRes-S. One reason why this did not lead to a difference in performance for most listeners is that the spread of excitation is probably more blurred than the electrodograms, due to current spread within the cochlea and, possibly, by neural factors.

It can also be seen that a local cue is introduced by the Optima-S strategy: The envelopes in channels 1–3 and 14 are asymmetric, and the direction of the asymmetry is different for up and down glides. Hence, in principle, the Optima-S strategy introduces a within-channel cue that listeners could have exploited. Unfortunately, attempts to remove the cue by setting the T and C levels in these edge channels to zero were only partially successful, with the asymmetry shifting to the new edge channels. Further attempts with listener AB24 to mask the asymmetries using modulated sine tones produced inconclusive results. During these additional listening sessions, the initial conditions were also retested (Figure 2, black and blue triangles). The repeat measurements of the Optima-S condition revealed much higher variability. There is the possibility that the cues AB24 is using at high thresholds are not related to the monotonically changing density/RPO cue, but to some other nonmonotonic cues that appear due to spectral aliasing of the stimuli due to the bandwidths of the filters used (conference abstract, O’Brien & Winn, 2017). It is also possible that higher nerve density in the cochlea of AB24 and AB26 enabled them to take advantage of the virtual channels provided by Optima™ current steering. Previous studies have shown that current steering improves the performance in spectral ripple tasks for some listeners (Drennan et al., 2010). However, there is too little evidence in this study to ascertain exactly what cues AB24 was using. In addition, the electrodograms do not show the interactions of current or the change in neural response when current steering is enabled, so observations of behavioral results combined with electrodograms are necessarily limited.

The fact that performance with the two strategies was similar for most listeners suggests that they did not use the cues available to AB24, although one cannot completely exclude the possibility that Optima-S somehow degraded the true spectro-temporal cue, and that use of the within-channel cue more-or-less exactly compensated for this. The improvement observed for AB24 may have been due to their overall high level of performance: Their excellent spectro-temporal resolution may have allowed them to exploit the improvement in the spectro-temporal representation of Optima-S or to have honed in on the extraneous within-channel cues.

## Conclusions

The effect of loudspeaker presentation, within- and across-channel envelope asymmetry cues, and processing strategy on the STRIPES test were investigated. Previous results were compared with results obtained from the same listeners using the new loudspeaker-presented version of the test. Listeners were tested using the same device and map as previously, and using their clinical devices and everyday program. No overall effect of presentation/test type or device/processing type was found. Therefore, the STRIPES test was robust to such manipulations and the nonspectro-temporal cues that were introduced such as the asymmetrical channel envelopes observed in the electrodograms (temporal only) and preemphasis (spectral-only weighting toward responding based on higher frequency channels). However, one listener obtained higher and more variable STRIPES thresholds using their clinical device relative to the other conditions tested. The source of this improved performance could not be clearly determined. A comparison of all published STRIPES thresholds to date showed that STRIPES produces generally robust and consistent thresholds for CI listeners across test sessions. The STRIPES test can be used as both a research and clinical tool to investigate listeners’ spectro-temporal resolution over a wide range of listening performance.
